# “It would be nice if the university appreciated the commitment more” – medical students and their learning and working experiences as co-caregivers during the pandemic

**DOI:** 10.3205/zma001778

**Published:** 2025-09-15

**Authors:** Christian Scheffer, Hagen Sjard Bachmann, Beate Stock-Schröer, Arndt Büssing

**Affiliations:** 1 Witten/Herdecke University, Faculty of Health, Integrated Curriculum for Anthroposophic Medicine, Witten, Germany; 2 Witten/Herdecke University, Faculty of Health, Institute of Pharmacology and Toxicology, Center for Biomedical Education and Research (ZBAF), Witten, Germany; 3 Witten/Herdecke University, Faculty of Health, Interprofessional Research School Integrative Medicine and Health Sciences IGIM, Witten, Germany; 4 Witten/Herdecke University, Faculty of Health, Professorship for Quality of Life, Spirituality and Coping, Witten, Germany

**Keywords:** workplace learning, COVID-19, undergraduate medical students, value added education, active student participation, collaboration, service learning

## Abstract

**Introduction:**

During the COVID-19 pandemic, many medical students played an active role in the healthcare sector. This cross-sectional survey was used to record the participants´ work and learning experiences.

**Methodology:**

During the first lockdown (May/June 2020), we conducted a nationwide online survey of medical students who took part in pandemic assignments.

**Results:**

A total of 381 medical students on placement participated in the survey. The most common placement locations were hospitals (60%), followed by outpatient care (21%) and the public health services (18%). Tasks included in particular nursing activities, blood sampling, patient consultations and smear tests.

Most students felt well integrated. Hospitals were associated with more stress and excessive demands.

The greatest learning gains were experienced in the areas of collaboration, communication, knowledge of the health system, practical skills, insights into everyday medical practice and crisis management.

In the free text responses, many respondents criticised the healthcare system particularly the focus on economics, bureaucratic processes and hierarchical structures. Positive feedback related to appreciation of nursing care.

Many students said that they missed support from the faculty. Looking to the future, many would like to see more practical and responsible activities in healthcare.

80% of participants would be available for such an assignment again.

**Conclusion:**

The integration of the students can be considered a success. Many experienced progresses in key areas of expertise and would like to see more structured active participation in healthcare.

## Zusammenfassung

**Einleitung:**

Während der COVID-19-Pandemie beteiligten sich viele Medizinstudierende aktiv in der Gesundheitsversorgung. Mit dieser Querschnittsbefragung wurde die Arbeits- und Lernerfahrungen der Teilnehmenden erfasst.

**Methodik:**

Während des ersten Lockdowns (Mai/Juni 2020) führten wir eine bundesweite Onlinebefragung bei Medizinstudierenden durch, die sich an Pandemie-Einsätzen beteiligten.

**Ergebnisse:**

Es beteiligten sich 381 im Einsatz befindliche Medizinstudierende an der Befragung. Die häufigsten Einsatzorte waren Krankenhäuser (60%), gefolgte von ambulanter Versorgung (21%) und dem öffentlichen Gesundheitsdienst (18%), die Aufgaben beinhalteten insbesondere pflegerische Tätigkeiten, Blutentnahmen, Patientengespräche und Abstrichentnahmen.

Die meisten Studierenden fühlten sich gut integriert. Belastung und Überforderung wurde in den Krankenhäusern höher erlebt.

Die höchsten Lerngewinne wurden in den Bereichen Zusammenarbeit, Kommunikation, Gesundheitssystemwissen, praktische Fähigkeiten, Einblicken in den medizinischen Alltag und Krisenmanagement erfahren.

In den Freitextantworten formulierten viele Kritik am Gesundheitssystem, insbesondere an der Ökonomisierung, an bürokratischen Prozessen und an hierarchischen Strukturen. Positive Rückmeldungen betrafen die Wertschätzung der Pflege.

Viele Studierende vermissten Unterstützung seitens der Fakultät. Für die Zukunft wünschen sich viele mehr Praxis und verantwortliche Tätigkeiten in der Gesundheitsversorgung.

80% der Teilnehmenden würden für einen solchen Einsatz wieder zu Verfügung stehen.

**Schlussfolgerung:**

Die Integration der Studierenden kann als ein Erfolg angesehen werden. Viele erlebten dabei Lernfortschritte in wesentlichen Kompetenzfeldern und wünschen sich mehr strukturierte Mitarbeit in der Gesundheitsversorgung.

## 1. Introduction

During the COVID-19 pandemic, the healthcare sector faced the extraordinary challenge of implementing comprehensive changes in many areas within a short timeframe in order to contain the further spread of the infection while caring for the sick. In preparation for the first wave in spring 2020, the focus in Germany was on providing more intensive care beds, postponing planned hospital procedures and ensuring hospital staff could work [1]. In this context, the Federal Minister of Health at that time Mr. Spahn, called for medical students to be deployed in healthcare. 

While many healthcare facilities employees were preparing for the arrival of the first wave and for extraordinary workloads, medical students found themselves in the opposite situation. Following the WHO’s declaration of a pandemic, social contact was restricted worldwide with face-to-face lectures and clinical internships being suspended, for example. This left many students underemployed with many expressing a desire for meaningful participation [2]. 

In this situation, numerous medical students decided to help deal with the pandemic by volunteering at various healthcare facilities. As part of the Medis-vs-COVID-19 volunteer initiative, around 20,000 medical students in Germany volunteered within a few weeks to help deal with the pandemic [3], [4]. Many students also volunteered through local organisations, while others increased their hours working in hospitals. 

The students were primarily motivated by altruism to support the healthcare staff, but were also interested in applying their more theoretical knowledge to improve their practical skills [5]. 

Using medical students to help manage the pandemic was a first in several respects:

- Traditionally, medical students are not employed as independent workers in the healthcare sector. Although participation in healthcare, e.g. in clinical clerkships or the practical year, is an important part of their training, they are primarily seen as learners and not as employees
- The deployment took place quickly and without much preparation time and in a dynamic situation. In many places capacity was increased (e.g. intensive care beds with ventilation options) or new facilities were established (e.g. testing centers).
- By participating, the students experienced the effects of the pandemic and the fight against it first-hand, including the associated uncertainties and difficulties.

While it makes sense to use medical students as a flexible and relatively safe workforce in an exceptional situation such as a pandemic, this raises important ethical questions. For instance, many students lack the necessary training and knowledge to respond effectively to a pandemic. Therefore, there was a call for hygiene training and appropriate supervision of the participating students [6]. In addition, such assignments, can be stressful so specific support in processing these experiences may be useful. 

This study aimed to find out how students experienced their assignments, particularly in terms of their integration as employees and the extent to which they found the assignment instructive for their career path. 

## 2. Method

We conducted an online cross-sectional survey among medical students during the first wave of the Coronavirus pandemic immediately after the first lockdown from May 29 to June 18, 2020. Participants were recruited via email distribution lists of the medical student councils and medical faculties in Germany, the Federal Representation of Medical Students in Germany and via Facebook groups such as Medis vs. COVID-19. After confirming their consent, students could complete the anonymous questionnaire (see attachment 1 ). Students could end the questionnaire at any time (see attachment 1 ) and were able to view and change their own answers. No incentives were offered. Approval was obtained from the Witten/Herdecke University ethics committee of (#106/2020). 

The survey comprised various standardised questionnaires on psychosocial issues, such as motivation, stress perception and well-being. The results of these questionnaires are presented in another publication [5]. In accordance with the research question, only the responses of the medical students in the field were evaluated for this publication.

In addition to items on socio-demographic items, the questionnaire contained questions about the work placement (type and duration) and items about stress (4 items, Cronbach’s alpha=0.77), the experience of excessive demands (3 items, Cronbach’s alpha=0.69), the experience of deficits (3 items, Cronbach’s alpha=0.64) and appreciation (3 items, Cronbach’s alpha=0.73). These were rated on a 5-point Likert scale.

Another set of questions covered the students’ learning experiences and was developed through an iterative cooperative process based on the CanMEDS roles [7]. These predefined skills were rated using a Likert scale ranging from 0 (strongly disagree) to 4 (strongly agree). 

SPSS 28.0 was used for the statistical analyses. The groups were compared using ANOVA. The effect sizes were quantified using Eta^2^.

Additionally, two free-text questions relating to further areas of learning and to wishes for future studies were asked. These free text responses were analysed using qualitative content analysis methods [6], with two student and two academic members of the working group independently coding and forming main and subcategories, and then merging them in an iterative process. MAXQDA 11 (VERBI Software, 2019) was used for the qualitative analysis.

## 3. Results

### 3.1. Participants and areas of application

A total of 731 medical students from all federal states with medical faculties took part in the survey: 19% from Hanover, 15% from Aachen, 9% from Giessen, 7% from Münster, 5% from Berlin, 5% from Munich, 40% from other faculties. Of these, 381 (52%) were in employment (see table 1 (Tab. 1)). 

The students' areas of work and responsibilities are shown in table 2 (Tab. 2). In all, 89% of the students stated that they received some kind of honorarium, 6% received nothing, 5% received meals and 1% were reimbursed for travel expenses.

### 3.2. Support in the workplace

68% received introductory training at the beginning of the assignment, 1% reported receiving regular training throughout their assignment, while 17% received materials for self-directed learning. Reflective discussions about the assignments took place in an informal setting: 72% discussed their assignment with employees, 71% with fellow students, 80% with friends, family or a partner and 3% used professional discussion opportunities.

### 3.3. Experiences in the field: workload, excessive demands, living with deficits and appreciation

17.6% of students reported experiencing time pressure, while 26.3% reported significant physical stress and 18.6% reported psychological stress. Excessive demands due to a lack of previous medical knowledge were rare (5.5%), whereas a lack of practical experience was slightly more common (13.2%). 22.3% of students felt affected by the personal fate of patients. A lack of staff (35.9%) and good communication structures (35.4%) were common, and in some cases the area of activity was also unclear (28.5%). A smaller number of students felt useless (10.6%) or as though they were being used as cheap labour (16.9%). 

69.5% felt that they were taken seriously as employees, with a further 19.2% feeling this to some extent. The vast majority were at least able to ask questions (86%), and 67.5% were able to contribute ideas and suggestions in the workplace.

Overall, workload, excessive demands and the experience of deficits were rated rather low and appreciation was moderate (see table 3 (Tab. 3)). There were only slight gender-related differences in the experience of excessive demands, which were reported less frequently by men than by women. The stage of their studies preclinical or clinical) had no significant impact on students’ experiences in the workplace. The place of employment was more significant: those who were employed in inpatient care experienced greater stress and excessive demands. There were no relevant differences in experiencing deficits. Appreciation was comparatively high. 

### 3.4. Learning gains 

Collaboration was experienced as a significant learning gain across all locations. In hospitals, practical skills and medical routines were also highly rated, while in the public health service crisis management, communication and health system knowledge were highly rated. 

A high level of confirmation of the medical profession was reported in both inpatient and outpatient roles, and to a slightly lesser extent in the public health service. Across all deployment areas, there was a high willingness to sign up for such a deployment again if required (see table 4 (Tab. 4)).

### 3.5. Free text answers

The qualitative analysis of the free text responses regarding the other learning areas revealed that they could be categorised into four groups (see attachment 2 and table 5 (Tab. 5)).

#### 3.5.1. The healthcare system

Students share their insights into the healthcare system and the processes of various institutions. They complain about the public health service’s bureaucratic and inflexible processes, and the lack of cooperation between the various stakeholders in the healthcare system. The strong economisation of hospitals is criticised frequently. There are also several mentions of concerns about the efficiency of the healthcare system in Germany.

#### 3.5.2. Collaboration within the healthcare team

Students describe their diverse experiences and learning progress when working on site. This includes acquiring practical skills and implicit knowledge of work processes and documentation tasks in their respective areas. 

Hierarchical structures and interprofessional conflicts are viewed as obstacles to collaboration, whereas respectful collaboration and equal partnership are considered beneficial. The work of nursing is appreciated several times. Communication skills and crisis management in exceptional situations are frequently mentioned as areas for learning.

#### 3.5.3. Self-awareness and self-development

Several students said that the work pushed them to their limits and that they had to learn to take care of themselves. They also said t that they learned to face crises in a more relaxed and sober manner. Dealing with ethical issues in exceptional situations is an important topic. The assignment was characterised as motivating and meaningful for their studies. 

#### 3.5.4. Support for the commitment

Some students complained in their comments about inconsistent regulations at their universities regarding how studies and examinations should be conducted during the pandemic. Several students expressed their disappointment that voluntary commitment was not rewarded, but instead led to disadvantages.

The answers regarding future study preferences and wishes for future studies can be divided into two categories: 

#### 3.5.5. More responsible involvement in practice

By far the most frequently mentioned answers relate to the desire for the degree course to be more practical. This is based on the deficits experienced in various care settings, as well as the advantages of being part of care teams with concrete responsibilities and interprofessional collaboration. 

#### 3.5.6. Concrete curricular proposals

Based on their experiences during the pandemic, the students make specific suggestions for improving preparedness for their respective activities or for incorporating such content more frequently into their studies. As well as disaster management, intensive care medicine and public health services, this also includes preparing for challenging situations.

## 4. Discussion

The situation of medical students in Germany during the pandemic has been studied several times. For instance, the negative impact of the pandemic on the mental health and quality of life of medical students has been documented [6] as have increased stress levels resulting from due to social isolation and uncertainty regarding future studies [8]. While the switch to digital learning was appreciated by the students [9], it could not compensate for essential aspects such as social contact with fellow students and lecturers and practice in practical training [2], [10], [11]. 

By contrast, comparatively little is known about how students were involved in the pandemic in Germany. In an online survey conducted shortly before ours (April/May 2020) [12], medical students expressed satisfaction with their work and supervision. However, their more detailed work experiences and learning progress they experienced have yet to be investigated. 

Our survey was completed by medical students from all over Germany; the average age and predominance of women are both typical of medical students. More than a third of those involved had previous training in a healthcare profession, which undoubtedly lowered the threshold for participation. The most common places of employment were hospitals and the public health service, which expanded significantly during this time. Overall, this demonstrates the significant advantage of students being able to work flexibly in different areas.

The high level of physical, temporal and psychological stress experienced by some students was to be expected for such deployments and demonstrates the significant workload they were expected to undertake. The emotional impact of encountering patients' fates was also significant. According to the free text responses, this was due not only to the effects of illnesses caused by the virus, but also to the social restrictions experienced, for example when relatives were not allowed to visit their sick relatives. Overall, more opportunities for reflection and psychological support would certainly have been helpful in dealing with the experienced stress. Such structured support is essential, not only to reduce the participants' stress, but also to help them learn how to to deal with ambiguity, uncertainty and complexity, and to promote their personal development [13], [14].

Appropriate fields of activity, preparatory training and professional involvement under appropriate supervision are essential for deploying students in healthcare. According to our results, most students received training during their assignments. They rarely felt overwhelmed or useless; although the area of work was sometimes unclear. This may have improved over time, however [15].

One issue to note is the misuse of students as cheap labour in such situations, as reported by one in six students. This issue also emerged in the free text responses, when students perceived themselves as being at the bottom of the hierarchy and got the impression that they were being used not for unexpected emergencies, but to compensate for structural deficiencies. However, most students felt that they were taken seriously as employees who could contribute their own ideas and suggestions. 

“COVID-19 shows us our problems like through a burning glass.” This well-known saying can also be applied to the students’ views of the healthcare system. Students have criticised the lack of flexibility and bureaucratic processes, as well as the strong focus on economics and impersonal atmosphere, especially in larger hospitals. This fundamental criticism is in line with previous studies, which found that the increasing economisation of medicine was a significant burden for students [16]. 

In recent years, many initiatives in medical studies internationally and nationally have focused on identifying other areas of competence that are essential for the medical profession in addition to traditional specialist expertise and integrating these into medical training in a structured manner. For example, based on the CanMEDS role model, communication, collaboration, health advocacy and professionalism have been integrated. Our survey suggests that students gained significant learning experience in these areas during their placement. Similar experiences have been reported in the Netherlands [17]. An Irish study [18] also highlights communication, teamwork, compassion and altruism as key learning areas, while a qualitative study from Heidelberg [14] points to the importance of placement in forming a professional identity. 

The qualitative statements clearly show that the participants would have liked more flexibility and support from faculty members. Many also expressed their desire for their studies to be more practically relevant by working responsibly in teams. Against this backdrop, the question arises as whether medical students can be integrated into healthcare in a way that enables them to learn and contribute simultaneously. This has not only taken place during the pandemic, but also in other situations of shortage, e.g. in caring for the homeless or in disaster prevention. An international movement for *“value-added education”* [19], [20], [21] has emerged in the USA, where education also generates added value for care. In this sense, the Corona-related tasks assigned to medical students can also be viewed as offering a glimpse into the future, where students actively participate in healthcare and gain invaluable insights into their professional development.

### 4.1. Limitations

The advantage of online surveys is that a large number of people can be surveyed in a short amount of time with relatively little effort. A major disadvantage, however, is the typically low response rate. It is unclear how many of the 108,000 medical students enrolled in Germany participated in pandemic operations. However, if we compare our study with data from the nationwide Medis-vs-COVID-19 network, which has more than 20,000 students, of whom more than 10,000 were deployed, there is no significant difference in terms of gender, age or semester. The representation of the vast majority of federal states also suggests that the data is representative.

When evaluating the survey results, bear in mind that these are student´s feedback rather than objective examination results or external assessments.

## 5. Conclusion

A major crisis, such as a pandemic, creates a high level of pressure for change. It therefore presents an opportunity to break new ground and implement fundamental changes. In terms of education, online learning has particularly benefited from the pandemic. Our results suggest that there is further potential for development: namely, integrating medical students into healthcare so that they can contribute to healthcare while learning essential skills for their future profession. From the perspective of the medical students who took part in our study, appreciation, a flexible study design and appropriate learning support are essential for the successful involvement of students in healthcare. Further studies are needed to research how such “learning through co-care” could be developed in medical studies.

## Acknowledgements

We would like to thank the CoronAid group for their commitment in organising student support at the University of Witten/Herdecke, particularly the members who were involved in the qualitative and quantitative evaluation: the students Anna di Bari, Oliver König and Alexander Lindeberg. 

## Authors’ ORCIDs

- Christian Scheffer: [0000-0003-0350-707X]
- Beate Stock-Schröer: [0009-0007-3537-3287]
- Arndt Büssing: [0000-0002-5025-7950]

## Competing interests

The authors declare that they have no competing interests. 

## Supplementary Material

Questionnaire for students in action during the COVID-19 pandemic

Qualitative analysis of learning and work experiences

## Figures and Tables

**Table 1:**
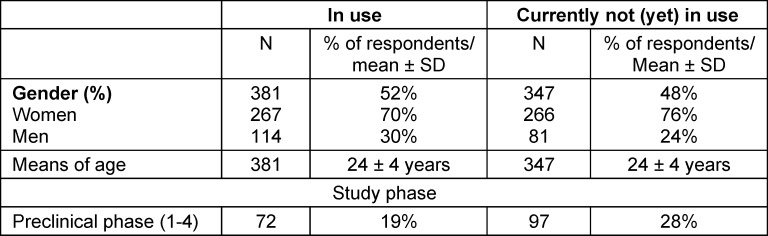
Description of participants (n=731; in use: 381)

**Table 2:**
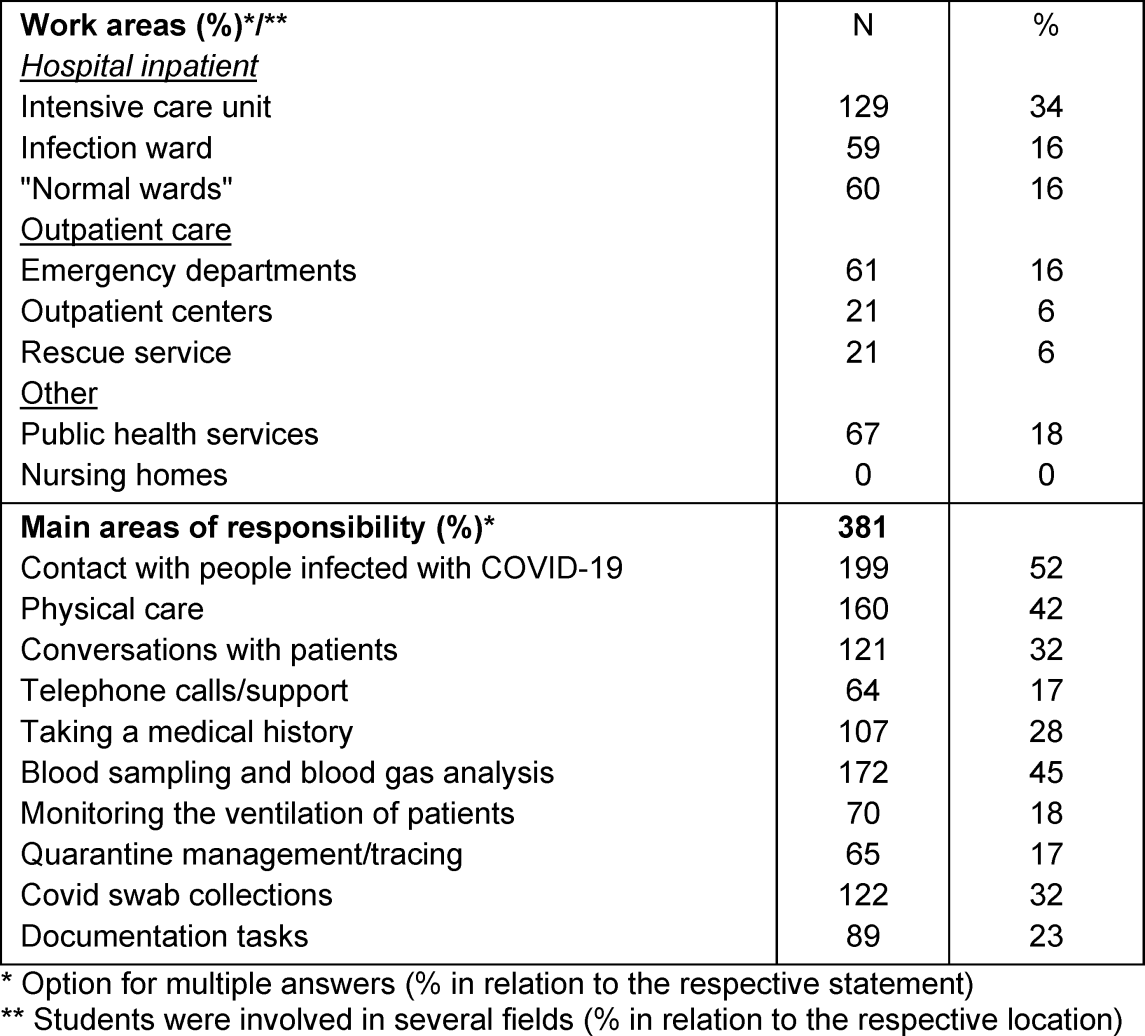
Areas of work and areas of responsibility of the employees (n=381)

**Table 3:**
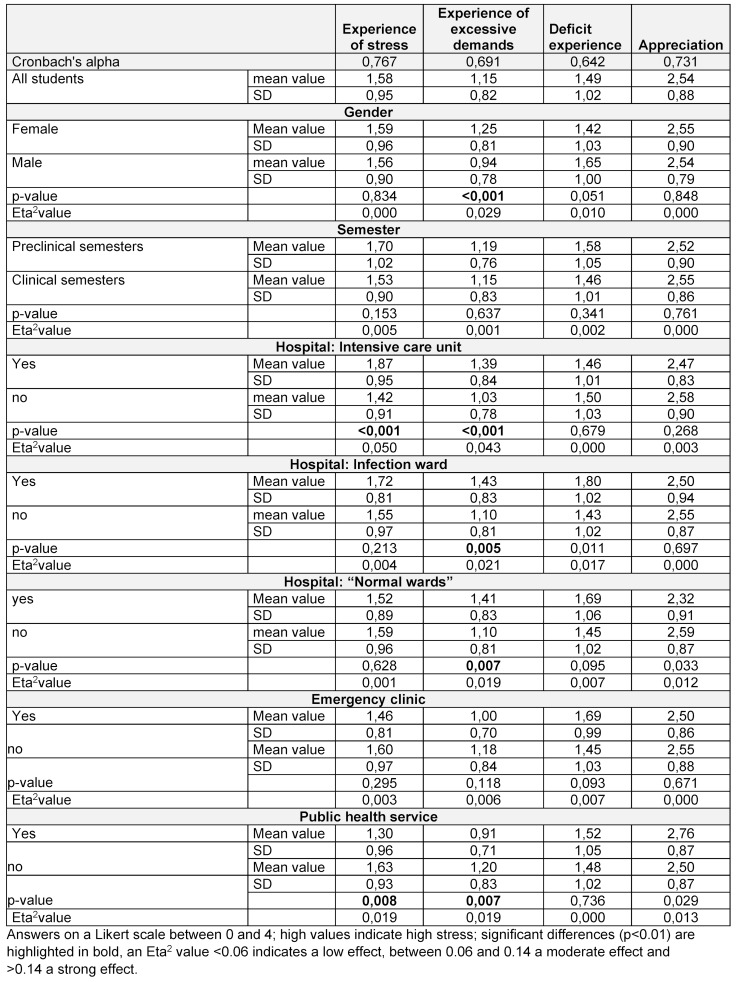
Experiences during the assignment in relation to the location, gender and semester of the students

**Table 4:**
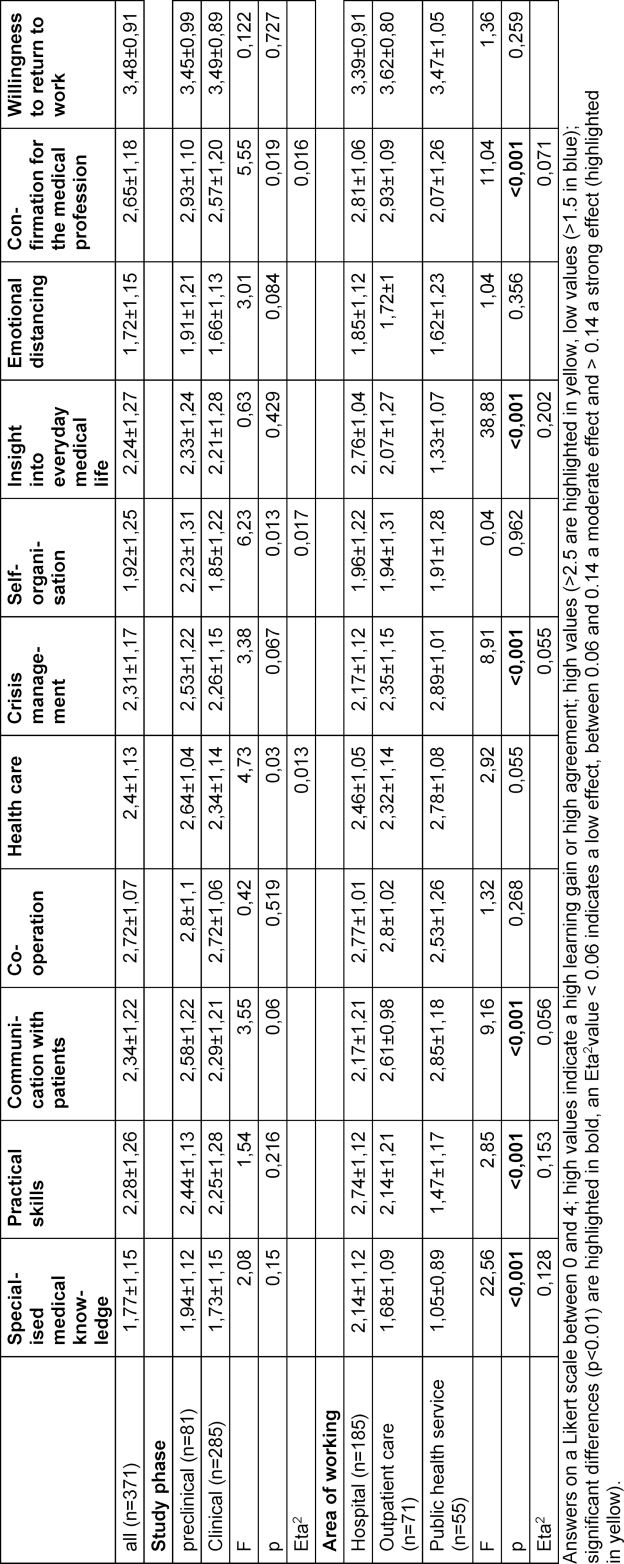
Subjective learning gain in relation to the study phase and area of application

**Table 5:**
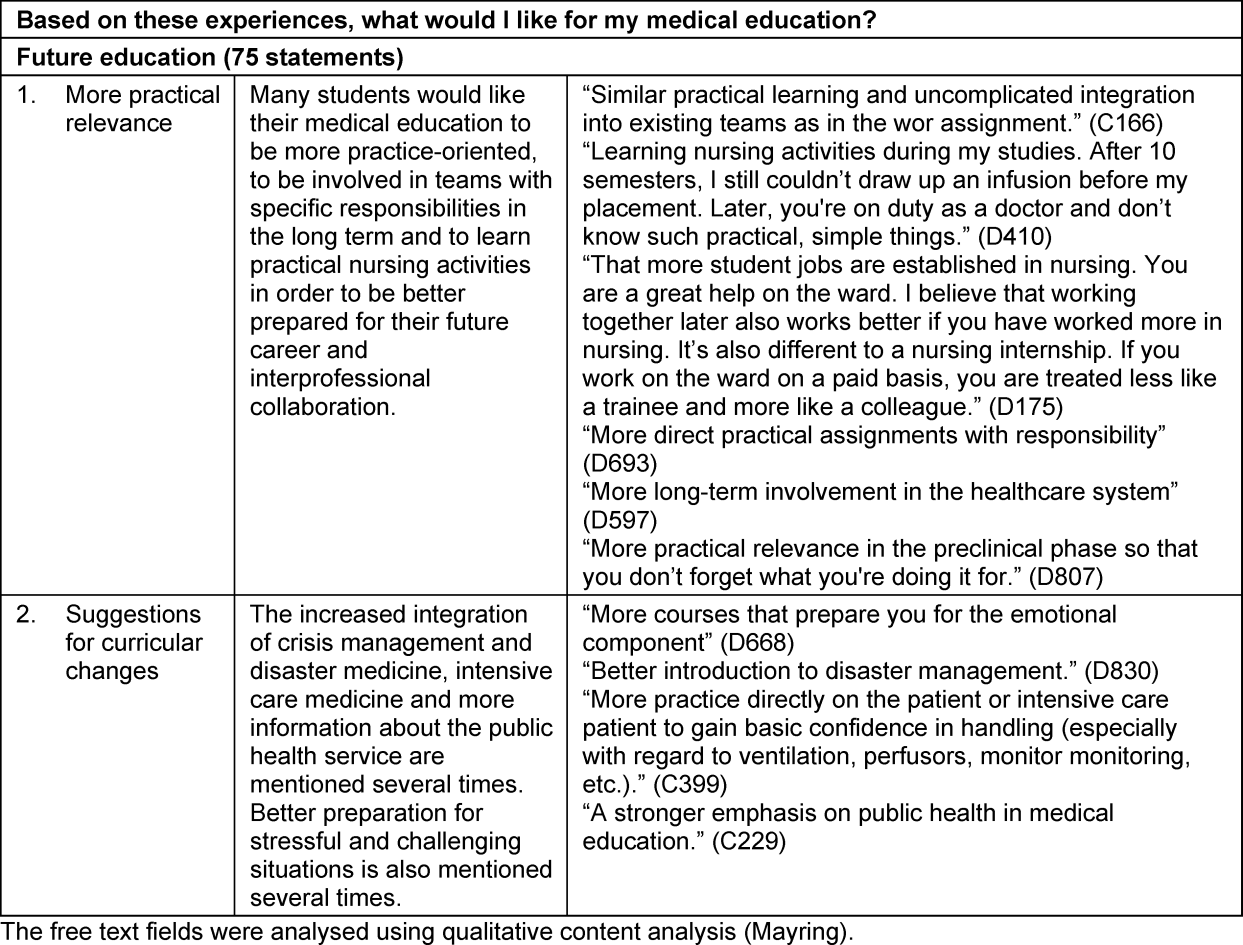
Qualitative analysis of the free texts on future studies
