# Supplementary material for: “It would be nice if the university appreciated the commitment more” – medical students and their learning and working experiences as co-caregivers during the pandemic
Source: GMS J Med Educ. 2025 Sep 15;42(4):Doc54. doi: 10.3205/zma001778 (PMC12527396; doi:10.3205/zma001778)
Supplement: Questionnaire for students in action during the COVID-19 pandemic [file JME-42-54-s-001.pdf]

Attachment 1: Questionnaire for students in action during the COVID-19 pandemic

Dear students.

We are conducting a nationwide survey at all medical universities to evaluate the motivation, experience, perceived stress and available resources of students in the current situation, in which they are also required to work in healthcare.

We would like to invite you to take part in this **anonymous and voluntary survey**. It will take you about 30 minutes - yes, this seems to be a long time. However, it is a good opportunity to reassure yourself of your motives and behavior. There are no "right" or "wrong" answers, your individual experience is of relevance.

All data collected during the survey will be treated **as strictly confidential** and will only be processed in anonymized form. It is expressly emphasized that all information you have provided here can no longer be attributed to you as a specific person. The requirements of confidentiality and data protection are fully respected.

Additional comments on data protection:

No personal information about you is stored in the survey responses. IP addresses of respondents are not logged. Accordingly, no statements can be deleted retroactively. Timings are not saved; however, the answers are provided with a time stamp. Google Analytics settings: None

Socio-demographic data

Age groups: O< 20   O 20-25   O 26-30   O >30
Gender: m/f/d
University: ....
Semester: ....

Have you completed vocational training that qualifies you for an assignment?
O Paramedic / rescue assistant / rescue helper
O Health and nursing care
O Medical assistant
O Medical-technical (laboratory) assistant
O Other healthcare professions: ....

Information about your appointment

I am already or have already been deployed:
O applies   O does not apply

I have registered for an assignment, but have not yet been assigned:
O applies   O does not apply

Attachment 1 to Scheffer C, Bachmann HS, Stock-Schröer B, Büssing A. *"It would be nice if the university appreciated the commitment more" – medical students and their learning and working experiences as co-caregivers during the pandemic.* GMS J Med Educ. 2024;42(4):Doc54. DOI: 10.3205/zma001778

I have not registered and do not want to be deployed:
O applies O does not apply
→ If you have answered YES here, you do not need to fill in the information on the assignment, please first fill in the information on workload and resources again

How many hours are/were you on duty per week? O 10 O 20 O 30 O 40 h

How long have you been in the field?
O I am not yet working O for approx. 1 week O for approx. 2 weeks O for more than 2 weeks

I am currently working: yes / no

How do you feel about the amount of time you spend at work? O too high O balanced O too low

I would sign up again as a student for such an assignment in healthcare at any time.
O strongly agree, O somewhat agree, O partly/partly agree, O somewhat disagree O strongly disagree

Where will you be deployed?

O Medical practice/office/MVZ	O Health authority and other official
O Hospital: Intensive care unit	O Hospital: Infection ward
O Hospital: emergency outpatient clinic	O Hospital: "normal wards"
O Care facilities / retirement home	O Rescue service
O Home visits	O Other:______

What are your main tasks? → Multiple answers possible

- Contact with COVID-19 sufferers
- Physical care / positioning of patients or nursing home residents
- Conversations with patients or care home residents
- Taking a medical history and physical examination of patients
- Blood sampling and blood analysis
- Monitoring the ventilation of patients
- Swab collection
- Quarantine management / tracing of contact persons
- Data entry / recording of statistics
- Telephone calls / psychological support
- Other: ....

How do you experience your work
O strongly agree, O somewhat agree, O partly/partly agree, O somewhat disagree O strongly disagree

During my assignment...

- ...I was under time pressure
- ...I felt physically burdened
- ...I felt psychologically burdened
- ...I felt overwhelmed by my lack of medical knowledge
- ...I felt overwhelmed by my lack of practical experience
- ...I felt personally affected by the fates of the patients
- ...there was a shortage of staff
- ...lacked good communication structures
- ...my field of activity was not clearly defined
- ...I felt useless
- ...I felt abused as cheap labor
- ...I feel that I am taken seriously as an employee
- ...I was able to contribute well with my ideas and suggestions
- ...I was able to ask good questions

What opportunities for discussion have you used regarding your assignment?

- ...discussions with employees in the field yes / partly - partly / no
- ...conversations with fellow students yes / partly - partly / no
- ...conversations with partners / families yes / partly - partly / no
- ...Professional discussion offers yes / partly - partly / n

Motivation

Please use the 7-point scale to assess what motivated you to get involved in providing care during the coronavirus pandemic.

Not at all applicable 1 2 3 4 5 6 7 Very applicable

- Interest in the clinical side of the pandemic
- Practical application of theoretical knowledge
- Knowledge gain for me
- Good for my professional qualification or CV
- Crediting as academic achievement
- Financial
- To get better into the role of a helper in the healthcare system
- To get better into the medical role
- To be able to contribute to overcoming the crisis
- To be able to stand up for society
- When sick people are in such existential need, you have to help
- When doctors and nurses are under such pressure, you have to help
- Some of my friends / acquaintances are also involved
- I can do something to escape my own worries
    - Medical students should help deal with real health problems
    - There should be more opportunities for such assignments in medical studies.

Attachment 1 to Scheffer C, Bachmann HS, Stock-Schröer B, Büssing A. *"It would be nice if the university appreciated the commitment more" – medical students and their learning and working experiences as co-caregivers during the pandemic.* GMS J Med Educ. 2024;42(4):Doc54. DOI: 10.3205/zma001778

Assistance from the

What support was provided by the institution before and during the assignment?

- Did the institution provide training? O yes O no O partially
- Did the institution provide teaching materials for independent learning? O yes O no O partially
- Did the institution provide training? O yes O no O partially
- Was there a permanent contact person at the facility? O yes O no O partially

- Is there a fixed contact person among the doctors? O yes O no O partially
- If yes, do you have the opportunity to discuss and reflect on your assignment with them? O yes O no O partially
- Were wishes, such as the definition of working hours, taken into accountO yes O no O partially
- Were concerns and fears communicated openly within the team?O yes O no O partially
- Were your problems addressed? O yes O no O partially
- My resilience limits and skills I have not yet acquired were taken into account. O yes O no O partially
- I felt valued by the organization during your work. O yes O no O partially
- I felt welcomed by other people in the team. O yes O no O partially

If there was training in the facility, how was it organized?
O One-off training at the beginning of the assignment O Regular training throughout the entire assignment O Materials provided for independent learning

Skills acquisition

What do you learn from the assignment?
O strongly agree, O somewhat agree, O partly/partly agree, O somewhat disagree O do not agree at all

- I learn a lot of medical knowledge
- .I learn many practical skills
- I learn a lot about communicating with patients, relatives, nursing home residents, etc.
- I learn a lot about collaboration
- I learn a lot about health care
- I learn a lot about crisis management
- The additional work I did during my studies taught me how to organize myself well
- The assignment gave me a very good insight into everyday medical life
- Through the assignment, I learned to distance myself more in order to be able to do my job

Burdens and ressources

	The following questions deal with how often you felt stressed during the last month	never	rare	sometimes	frequently	very often
1	How many times in the last month have you been upset that something completely unexpected has happened?	0	1	2	3	4
2	How often in the last month have you had the feeling that you couldn't influence important things in your life?	0	1	2	3	4
3	How often have you felt nervous and "stressed" in the last month?	0	1	2	3	4
4	How often in the last month have you felt confident in dealing with personal tasks and problems?	0	1	2	3	4
5	How often in the last month have you had the feeling that things are going your way?	0	1	2	3	4
6	How often in the last month have you had the feeling that you couldn't cope with all the tasks and problems at hand?	0	1	2	3	4
7	How often in the last month have you felt like you were coping with anger in your life?	0	1	2	3	4
8	How often in the last month did you feel like you had everything under control?	0	1	2	3	4
9	How often in the last month have you been annoyed about not being able to influence important things?	0	1	2	3	4
10	How often in the last month have you had the feeling that your problems have piled up so much that you can no longer cope with them?	0	1	2	3	4

PSS © Cohen; Eng. Version: Arndt Büssing, University of Witten/Herdecke

The following statements relate to your well-being in the last 2 weeks. For each statement, please mark the section that you think best describes how you have felt in the last 2 weeks.

In the last 2 weeks ...	All the time	most	Just over half the time	A little less than half the time	Now and then	At no time
... I was happy and in a good mood	5	4	3	2	1	0
... I felt calm and relaxed	5	4	3	2	1	0
... I felt energetic and active.	5	4	3	2	1	0
... I woke up feeling fresh and rested.	5	4	3	2	1	0
... my everyday life was full of things that interest me.	5	4	3	2	1	0

WHO-5 © Psychiatric Research Unit, WHO Collaborating Center for Mental Health, Frederiksborg General Hospital, DK-

Use the following answer options to indicate whether or how often you experience the following physical complaints. There are no right or wrong answers. Please do not skip any question! → Period: Use

O Never – O every few months – O every few weeks – O every few days – O almost daily

- Do you tire quickly?
- Do you have a headache?
- Do you feel it all over your body when you are upset about something?
- Do you have back pain?
- Are you nervous?
- Do you have neck pain?
- Do you have shoulder pain?
- Do you have concentration problems?
- Do you have trouble sleeping (difficulty falling asleep, trouble sleeping through the night)?
- Do you feel tired and exhausted throughout the day?

**Dealing with yourself and others**	
Please answer how **often** you notice the above feelings and reactions during your deployment (or during your training, if you are not yet deployed or have decided against it, for example) when you meet patients.<br>1 = a few times a year or less; 2 = once a month; 3 = a few times a month; 4 = once a week; 5 = a few times a week; 6 = daily	
When dealing with the patients I have to care for, I notice that	**How often?** (1-6)
...that I simply can't let everything get so close to me anymore.	
...that I have to take a step back more and more often to protect myself.	
...that their personal problems and worries are often just too much for me.	
...that I often no longer have the patience to listen to them.	
...that I don't really care what they think of me.	
...that I'm thinking more and more often about just throwing everything away.	
...that some of them just annoy me.	
...that I'm increasingly missing out myself.	
...that I am increasingly doing "work to rule".	
...that I have to keep a clear distance in order to protect myself.	
...that I think of them every evening in a devout review of the day.	

CDI © Arndt Büssing, Witten/Herdecke University

Please answer the following questions honestly and as quickly as possible using the following polarized rating scale:<br>not really applicable [1 2 3 4 5 6 7] strongly agree									
C1	When I hear about someone (a stranger) who is going through a difficult time, I feel great compassion for him or her.	0	1	2	3	4	5	6	7
C2	I tend to have compassion for others, even if I don't know them.	0	1	2	3	4	5	6	7
C3	One of the activities that is most important to me in life is helping others in the world when they need my help.	0	1	2	3	4	5	6	7
C4	I would rather choose actions that help others (even if they are strangers) than stand up for things that help me.	0	1	2	3	4	5	6	7
C5	I often have loving feelings for people (strangers) when they seem to be in need.	0	1	2	3	4	5	6	7

Santa Clara Brief Compassion Scale © Hwang, Plante & Lackey

Please describe here how often you have the following feelings and experiences		never	rare	frequently	regularly / very
ED1	I am often overcome with a feeling of great gratitude.	0	1	2	3
ED2	I am often overcome by a feeling of amazement and awe.	0	1	2	3
ED3	I have experienced and learned to appreciate beautiful things.	0	1	2	3
ED4	I often stop and am mesmerized by the beauty of nature.	0	1	2	3
ED5	I often pause and stay spellbound in the moment.	0	1	2	3
ED6	In certain places I become very quiet and reverent.	0	1	2	3
ED7	I often stop and think of so many things for which I am truly grateful.	0	1	2	3

GrAw-7 © Arndt Büssing, Witten/Herdecke University

Attachment 1 to Scheffer C, Bachmann HS, Stock-Schröer B, Büssing A. *“It would be nice if the university appreciated the commitment more“ – medical students and their learning and working experiences as co-caregivers during the pandemic.* GMS J Med Educ. 2024;42(4):Doc54. DOI: 10.3205/zma001778

Current behavior:

Smoking ☐ never☐ 1-10 cigarettes a day☐ 10-20 cigarettes a day☐ more than 20 cigarettes

Alcohol ☐ never☐ 1x per month☐ 2-3x per month☐ 1-2x per week☐ several times per week

Sport ☐ never☐ 1x per month☐ 2-3x per month☐ 1-2x per week☐ several times per week

Meditation:☐ never☐ at least 1x per month☐ at least 1x per week☐ at least 1x per day

Pray: ☐ never☐ at least 1x per month☐ at least 1x per week ☐ at least 1x per day

Performance-enhancing medication:☐ never☐ at least 1x per month☐ at least 1x per week☐ at least 1x per day

Sedative medication:☐ never☐ at least 1x per month☐ at least 1x per week ☐ at least 1x per day

What would I wish for from these experiences for my medical studies?

Many thanks for the effort
We wish you much success and good health in your studies!

Attachment 1 to Scheffer C, Bachmann HS, Stock-Schröer B, Büssing A. *“It would be nice if the university appreciated the commitment more“ – medical students and their learning and working experiences as co-caregivers during the pandemic.* GMS J Med Educ. 2024;42(4):Doc54. DOI: 10.3205/zma001778
