# Supplementary material for: “It would be nice if the university appreciated the commitment more” – medical students and their learning and working experiences as co-caregivers during the pandemic
Source: GMS J Med Educ. 2025 Sep 15;42(4):Doc54. doi: 10.3205/zma001778 (PMC12527396; doi:10.3205/zma001778)
Supplement: Qualitative analysis of learning and work experiences [file JME-42-54-s-002.pdf]

Attachment 2: Qualitative analysis of learning and work experiences

If you have learned something else from the task, what is it?		
**Healthcare system (25 statements)**		
**Category**	**Explanation**	**Examples**
1. the healthcare system as a whole and cooperation between the individual stakeholders	Cooperation between the individual stakeholders is perceived as inflexible and ineffective. Concerns about the performance of the healthcare system during the pandemic.	"The communication between the doctors in outpatient care, the test centers and the health authorities did not and does not work at all." (D8)<br>"The structures are fixed and entrenched." (C685)<br>"The German system would not be able to cope with a pandemic outbreak with an exponential increase in patients requiring medical treatment, especially in intensive care units." (D13)
2. work in hospitals	Healthcare is perceived by some as impersonal because hospitals are too large, commercially oriented and uncoordinated.	"That hospitals are more focused than ever on financial turnover and figures. The patient is no longer the decisive factor. The main thing is that they have the "right" illness!" (D11)<br>"In small hospitals, they take more care of you and treat you as part of the team and not as unnecessary ballast." (D15)<br>"How chaotic things are in large hospitals. How little interest the administration takes in what's happening on the wards." (D66)
3. work in the public health service	They get to know working in the public health service, but some find it unattractive for their future career.	"That I would never want to work in a health department for a long time. For a short time, it was a great job with a good salary." (C147)<br>"How health authorities work together." (D48)
**Active participation in healthcare team (58 statements)**		
**Category**	**Explanation**	**Example**
1. practical skills and tacit knowledge	Practical activities such as performing ECGs, taking smears and hygienic behavior are learned and implicit knowledge is acquired, e.g. about everyday life in the intensive care unit, aspects of crisis management and documentation tasks.	"Everyday medical practice in the intensive care unit." (D7)<br>"Assessment of patients' needs for outpatient treatment and hospitalisation." (D26)<br>"Working under hygienic conditions." (C410)<br>"Learning to set priorities. Triage of critically ill patients in a major emergency situation." (C1111)<br>"Getting to grips with files. Writing letters." (C354)
2. teamwork and interprofessional practice	The students describe key aspects of successful (low-hierarchical, appreciative, committed) and unsuccessful collaboration. The importance of interprofessional collaboration at eye level and appreciation for nursing is emphasized.	"Dealing with the different personalities of colleagues." (D63)<br>"How to behave as a supervisor, especially as a senior physician, and what advantages a good leader has for a team." (D66)<br>"Hierarchical structures do not help to improve the situation." (D70)<br>"The work of nurses is completely underestimated. They are the ones who keep the intensive care units running." (D24)<br>"For the mutual recognition at eye level in the later cooperation between us doctors and the nursing staff, it was once again a very profitable experience for me." (D48)

Attachment 2 to Scheffer C, Bachmann HS, Stock-Schröer B, Büssing A. *"It would be nice if the university appreciated the commitment more" – medical students and their learning and working experiences as co-caregivers during the pandemic.* GMS J Med Educ. 2024;42(4):Doc54. DOI: 10.3205/zma001778

Active participation in healthcare team (58 statements)		
**Category**	**Explanation**	**Example**
3. communicative skills	Open-minded, de-escalating, authentic and respectful communication is seen as essential in everyday working life.	“De-escalating communication skills.” (D23)<br>“Start discussions with more openness.” (D27)<br>“Communication with difficult patients.” (D56)<br>“Spend time talking to patients, listen carefully.” (D75)
4. the role of the students in the team	Unclear roles and the experience of being at the bottom of the hierarchy are perceived as problematic, while a meaningful role and belonging to the team are perceived as motivating.	“The students are at the bottom of the hierarchy.” (D20)<br>“I found out that everyone had miscalculated and therefore so many people were permanently employed that there was simply no motivation and too little work.” (D36)<br>“Becoming part of a new team and feeling safe.” (D40)
**Self-awareness and self-development during the assignment (25 statements)**		
**Category**	**Explanation**	**Example**
1. borderline experiences, self-care and coping with critical situations	Borderline experiences in relation to their own time capacities or psychological stress as well as experiences of being able to deal with crisis situations more soberly and calmly.	“That I have to take good care of myself and if I don’t, nobody will.” (D9)<br>“Increasing my frustration tolerance.” (D23)<br>“See crisis situations and circumstances in a more sober and relaxed way.” (D42)
2. professionalism and medical ethics	Students describe attitudes and critical aspects of medical ethics, e.g. in specific pandemic situations such as the separation of patients and relatives. Responsibility and meaningful activities are valued.	“Dealing with the dying and death in the palliative care unit.” (D62)<br>“I have learned to pay more attention to how patients feel.<br>I have learned to deal better with isolation and to respond to patients and their loneliness.” (C410)<br>“How many personal tragedies have taken place. Not through COVID-19 infection, but through the measures taken by the government.<br>Ex: A woman who lost all her hair because she could not cope with the fact that she was not allowed to see her husband for 4 weeks (he was in a nursing home) and then he died without the opportunity to say goodbye.” (C737)<br>“Taking responsibility for my patients.” (C830)
**Support for the mission (52 statements)**		
**Category**	**Explanation**	**Example**
1. necessary support from the faculty	Recognition of the assignment as a study achievement (e.g. as a clinical elective), adequate payment and a flexible study structure to better combine study and work	“It was very difficult at times to reconcile university and work. A rule has already been created that the exams are a free attempt, but that still doesn’t help you to pass them. The compulsory courses also often overlapped with the assignment. It would be nice if the university appreciated the commitment more.” (C975)<br>“Getting involved and making a contribution to society means disadvantages for studying.” (C53).<br>“That the practical placement is credited towards the medical degree and that the Dean of Studies supports the placement in healthcare in order to overcome organizational difficulties.” (C872)<br>“That you are paid reasonably.” (C788)

Support for the mission (52 statements)		
**Category**	**Explanation**	**Example**
2. support of the operation	Good organization of the assignment, accompanying supervision and digital learning opportunities are seen as helpful for an assignment	"Better organization in advance, I think it would be nice to be able to really help and not just be so alibi-like." (C631)<br>"Intervision/supervision would have made work easier, especially in such stressful situations." (C644)<br>"Face-to-face teaching is also very easy to implement online and offers a more flexible daily routine, especially in order to be able to pursue jobs." (C1004)
